# Optimization of Pre-Heat Treatment for Nitriding

**DOI:** 10.3390/ma14247766

**Published:** 2021-12-15

**Authors:** Matthias Steinbacher, Stefanie Hoja

**Affiliations:** 1 Leibniz-Institut für Werkstofforientierte Technologien—IWT, 28359 Bremen, Germany; steinbacher@iwt-bremen.de; 2 MAPEX Center for Materials and Processes, Universität Bremen, 28359 Bremen, Germany

**Keywords:** nitriding, pre-heat treatment, bainite, martensite, annealing, quenching and tempering, austempering

## Abstract

To achieve a core strength that meets the requirements during service life, components to be nitrided are subjected to a pre-heat treatment. Since a higher strength prior to nitriding also has a positive effect on the achievable strength in the nitrided layer, an optimization of the pre-heat treatment may lead to better service characteristics of nitrided components. For this purpose, different optimizations of pre-heat treatment were investigated on the nitriding and quenching and tempering steels EN31CrMoV9 and EN42CrMo4 (AISI4140). One strategy was a change of the austenitization temperature for EN31CrMoV9 from 870 °C to 950 °C in order to solve the coarse carbides of the as-delivered state and realize a finer distribution of the carbides in the quenched and tempered structure. This special treatment lead to a higher hardness compared to the conventional treatment. The second investigated pre-heat treatment variant was a bainitic treatment instead of quenching and tempering. The bainitic initial microstructure increased the diffusion depth compared to conventionally quenched and tempered specimens. In addition the maximum hardness of the nitrided layer, the core hardness was significantly higher on the specimens with the bainitic microstructure. During subsequent nitriding, however, the bainite is tempered and loses some of its hardness.

## 1. Introduction

In order to increase the strength and wear resistance in the surface layer of the components, machine parts are frequently nitrided [1]. In this process, the components are exposed to a nitrogen-containing atmosphere at temperatures of approx. 480–590 °C. The nitrogen-containing gases from the atmosphere react at the component surface to form diffusible nitrogen, which then diffuses into the component‘s surface layer. The increase in hardness during nitriding is essentially due to precipitation hardening by iron and alloying element nitrides formed in the diffusion zone when the relatively low solubility of nitrogen in ferrite is exceeded and/or alloying elements with high nitrogen affinity react with the nitrogen. Directly below the surface, where the nitrogen concentration is the highest, the precipitated nitrides form a closed layer, the so-called compound layer (also white layer) composed of either gamma prime (γ’) or epsilon nitrides (ε), which give the component not only higher wear resistance due to their high hardness but also improved corrosion resistance.

An increase in strength in the base material cannot be achieved during nitriding. Depending on the material‘s condition, a loss of strength can even be observed due to the tempering effect during nitriding [2] especially if nitriding is aiming for high nitriding depth achieved during deep nitriding processes. In order to achieve a core strength that meets the requirements, it is therefore necessary to conduct a pre-heat treatment before nitriding. In practice, components to be nitrided are therefore usually quenched and tempered.

In the quenching and tempering process, martensitic hardening is carried out first, followed by a tempering step. For this purpose, the microstructure is first fully austenitized. The actual hardening takes place during cooling by selecting the cooling rate in such a way that the diffusion-controlled transformation into near-equilibrium or equilibrium microstructures is suppressed. Instead, the transformation from austenite to martensite takes place by a diffusion-less mechanism. Since martensite is usually not only very hard but also brittle after quenching, it is tempered in a second heat treatment step to increase ductility [3]. Furthermore, tempering is necessary to increase the temperature stability of the microstructure so that significant changes in the microstructure and associated properties no longer occur during operation. The temperature during tempering can vary depending on the material and the application. When quenching and tempering is applied as a pre-heat treatment for nitriding, care must be taken to ensure that the tempering temperature is about 30–50 °C above the nitriding temperature in order to avoid further tempering during nitriding.

In recent years, austempering has developed into an alternative final heat treatment to quenching and tempering for various applications, since comparable hardnesses with better ductility can be achieved in bainite and, due to the process, fewer dimensional and shape changes occur than in classical hardening and tempering [4]. In austempering, the material is first heated to austenitizing temperature and then quenched to a material-dependent transformation temperature above the martensite starting temperature MS. This temperature is maintained in a salt bath or fluidized bed until the austenite is more or less completely transformed into bainite. Subsequently, cooling to room temperature can be carried out as quickly as desired.

The bainite structure consists of a carbon supersaturated ferrite whose body-centered iron lattice is tetragonally distorted by the excess carbon in solution, similar to that of martensite [5]. However, very fine carbide precipitates are formed immediately downstream of the primary bainitic ferrite formation [6], as a result of which the lattice distortion and the associated transformation-related volume changes are smaller than in martensitic hardening.

The hardness of the bainite increases the closer the transformation temperature is to the martensite start temperature MS in an isothermal transformation. However, somewhat lower hardness values are achieved in bainite compared to martensitic hardening. Compared to a quenched and tempered condition of the same hardness, the yield strength of bainitic microstructures is higher, especially when a completely bainitic microstructure is present [7]. The deformation capacity is also greater in the bainitic state than after quenching and tempering to the same hardness [7]. One advantage of bainitic treatment over quenching and tempering is that tempering is not required to achieve high toughness. The biggest disadvantages of austempering are the complex process control and the long treatment times. With modern sensor technology, however, it is possible to monitor the bainite content during the process so that bainitic microstructures can be produced selectively and economically [8,9,10].

In the present work, the pre-heat treatments quenching and tempering and austempering were investigated for the nitriding and tempering steels EN31CrMoV9 and EN42CrMo4, which are often used for nitriding. The microstructure was examined metallographically after pre-heat treatment and after nitriding and compared to describe differences in microstructure. Hardness tests were also carried out on the nitrided surface layer and the core. Although these do not provide classical material parameters, they can serve as an indication of the mechanical properties to be expected.

## 2. Materials and Methods

### 2.1. Materials

The frequently used nitriding and quenched and tempered steels EN31CrMoV9 (0.33% C, 0.21% Si, 0.47% Mn, 2.31% Cr, 0.16% Mo, 0.12% V) (Acciaierie Bertoli Safau, Pozzuolo del Friuli UD, Italy) and EN42CrMo4 (0.44% C, 0.29% Si, 0.82% Mn, 1.09% Cr, 0.23% Mo) (Georgsmarienhütte GMH, Georgsmarienhütte, Germany) were selected for the investigations.

### 2.2. Heat Treatment

Table 1 gives an overview of the performed heat treatments applied to the material in as delivered state (QT). Conventional quenching and tempering was carried out for two hours at an austenitizing temperature of 870 °C for the material EN31CrMoV9 and 850 °C for the material EN42CrMo4 followed by oil quenching (Ipsen, Kleve, Germany). Both materials were then tempered at 630 °C for two h. This relatively high tempering temperature was selected because treatment temperatures of approx. 480–590 °C are common for nitriding and nitrocarburizing, and the tempering temperature for quenching and tempering should be approx. 30–50 °C above the subsequent nitriding or nitrocarburizing temperature to avoid further tempering during the thermochemical heat treatment. For the Material EN31CrMoV9 another variant of quenching and tempering was carried out with an increased austenitization temperature of 950 °C instead of 870 °C. These samples were only examined with regard to their microstructure and and their temperature stability during long-term tempering. Long-term tempering was performed in a vacuum furnace (Nabertherm, Lilienthal, Germany). The tempering temperatures and durations can be taken from Table 2.

For austempering, both materials were first austenitized for 45 min at 850 °C in a salt bath (Durferrit type, GS540, Mannheim, Germany) and then quenched to the transformation temperature in a salt bath (Nabertherm type, AS140, Lilienthal, Germany). The transformation temperatures for austempering were estimated from the respective available isothermal TTT diagrams [11] and are about 20 °C above the respective martensite start temperature. For the material EN31CrMoV9 the transformation temperature was 400 °C and for the material EN42CrMo4 360 °C. The samples were kept at transformation temperature for one hour in each case, which should be sufficient for a complete bainitic transformation according to the TTT diagram. After one hour of soaking in the salt bath the samples were cooled to room temperature in air and aqueous washed with corrosion inhibitor (SurTec 042, Zwingenberg, Germany). The as bainitised state will be referred to as One-Step-Austempering (OSA) from here on.

Before nitriding the samples having been QT and OSA trovalized to activate the as heat treated surfaces of the samples prior to nitriding to avoid any diffusion barrier, and therefrom non-nitrided spots formed during the subsequent heat treatment.

Gas nitriding was carried out at 520 °C for 20 h in a retort furnace system with controlled nitriding potential (Nabertherm, Lilienthal, Germany). The nitriding potential was controlled to K_N_ = 0.8. Heating was performed under ammonia atmosphere (Westfalen AG, Münster, Germany) to activate the sample surface. Cooling after nitriding was also carried out with the addition of ammonia to avoid nitrogen effusion and the resulting denitriding at the end of the process.

### 2.3. Charakterization of the Samples

For the evaluation of the microstructure after heat treatment, a metallographic cross-section was taken from the specimens. Cutting was performed using a wire-erosion technique (Matra Fanuc Robocut, Neuhausen, Germany) in order to avoid heat effect. The microstructure was etched with Nital (3% alcoholic HNO_3,_ Carl Roth, Karlsruhe, Germany) as standard and documented with the light microscope (Leica, Wetzlar, Germany) as well as the scanning electron microscope (Tescan, Brno, Czech Republic). For the visualization of the carbide distribution after quenching and tempering, the microsection was etched according to Murakami (10 g Potassium hexacyanoferrate (III)/10 g NaOH/100 mL H_2_O, all reagents from Carl Roth, Karlsruhe, Germany) for 5 min at 50 °C. Vickers hardness testing was also performed on the cross-section. The core hardness was determined five times, and the average value and standard deviation were formed from the results.

## 3. Results and Discussion

### 3.1. As-Received Condition

**Figure 1:**
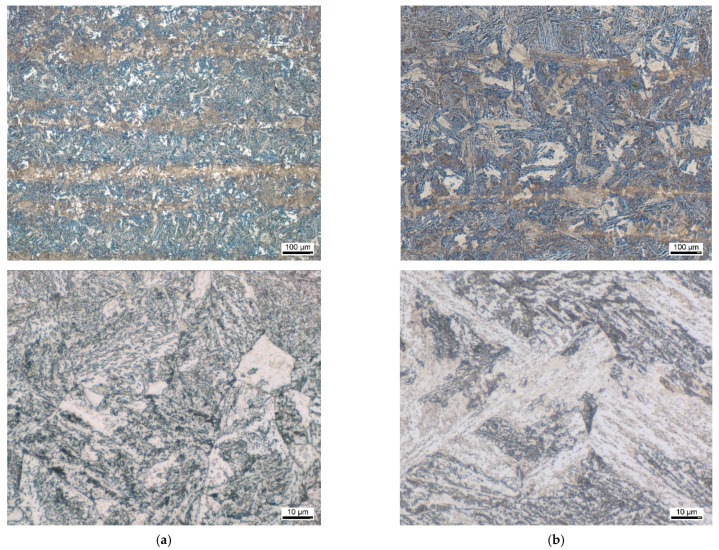
Light microscopic documentation of the as-received condition: (**a**) EN31CrMoV9, (**b**) EN42CrMo4.

Figure 1 shows the initial microstructure of both materials under investigation. In both cases, a highly quenched and tempered martensitic microstructure is present. The initial hardness is 353 HV10 for the material EN31CrMoV9 and 314 HV10 for the material EN42CrMo4. The microstructure exhibits an evenly distributed tempered martensite with a near equilibrium ferritic matrix and fine dispersed temper carbides. In the metallographic images of the cross section at lower magnification, some segregations related differences of the etching condition can be seen forming horizontal lined structures. These visible differences of the etching attack are usually related to an increased chromium content in the brighter areas, which somewhat weakens the etch attack and makes these areas appear brighter and less contrasted.

### 3.2. Quenching and Tempering

The size and distribution of the special carbides in the quenched and tempered structure is determined by the combination of the hardening temperature and the resulting solution state of the alloying elements and the subsequent tempering time and temperature. The temperature recommendations for hardening the material EN31CrMoV9 range between 870 °C and 930 °C, with the lower temperature range generally being selected in order to obtain the finest possible austenite grain. In the as-delivered condition of technical materials, precipitates of the alloying elements are usually already present, which are not dissolved during nitriding because the treatment temperature is low. Depending on the type of precipitates, they also may not be dissolved during preheat treatment due to the insufficient temperature and may even grow. The simulation software Thermo-Calc (Version 2017a, Stockholm, Sweden) was used to calculate the carbide content as a function of temperature for the material EN31CrMoV9. In the temperature range of 800 °C to 950 °C considered, only vanadium carbides are thermodynamically stable. Complete dissolution of these carbides is only achieved at temperatures above 885 °C.

**Figure 2:**
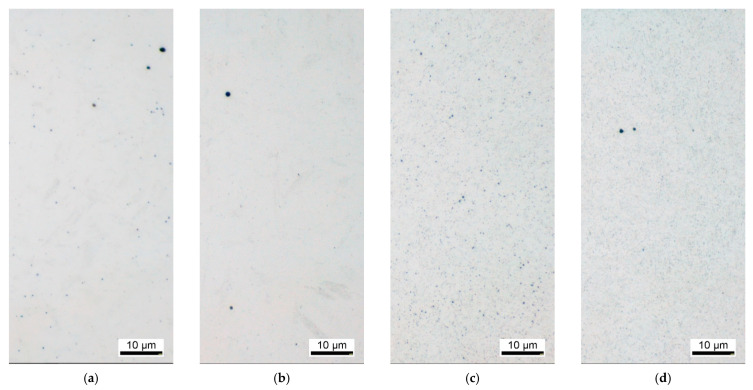
Precipitation distribution after quenching and tempering of the material EN31CrMoV9 of different austenitizing temperatures (etching according to Murakami to highlight the special carbides): (**a**) Quenched from T_A_ = 870 °C; (**b**) Quenched from T_A_ = 950 °C; (**c**) Quenched from T_A_ = 870 °C and tempered 2 h at 630 °C; (**d**) Quenched from T_A_ = 950 °C and tempered 2 h at 630 °C.

Figure 2 shows two precipitation states produced by different austenitizing temperatures, respectively after hardening and after tempering. The austenitizing temperatures were chosen to realize austenitization without complete dissolution of the carbides (T_A_ = 870 °C) and austenitization with complete dissolution of the carbides (T_A_ = 950 °C). At the lower austenitizing temperature of 870 °C, the carbides made visible by Murakami etching are relatively coarse after hardening. At 950 °C, the carbides are initially dissolved, and after hardening only isolated non-metallic impurities are visible. During the tempering treatment, fine carbides are again precipitated in both hardening structures, which can be seen as dark veils on the etched micrographs. In the case of the microstructure hardened and tempered at the usual temperature, the coarser carbides not brought into solution during austenitizing can also be seen.

**Figure 3:**
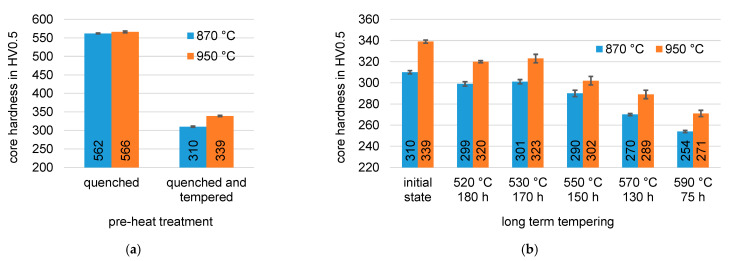
Core hardness after hardening of the material EN31CrMoV9 from different austenitizing temperatures (**a**) in the initial state and (**b**) after long-term tempering.

Figure 3 shows the results of the hardness test after quenching and tempering. In the hardened condition, both microstructures exhibit similar hardness. The finer carbide distribution after tempering of the 950 °C hardened condition leads to a slightly increased hardness. Both tempered microstructures were then subjected to different long-term tempering treatments to investigate the temperature stability of the pre-heat treatment microstructure. A decrease in hardness is observed with increasing tempering temperature for both tempered microstructures. However, the hardness difference between the two pre-heat treatment conditions is maintained during tempering, so that an increase in hardness in the nitrided layer and in the core can also be expected with regard to nitriding.

### 3.3. Austempering and Nitriding

**Figure 4:**
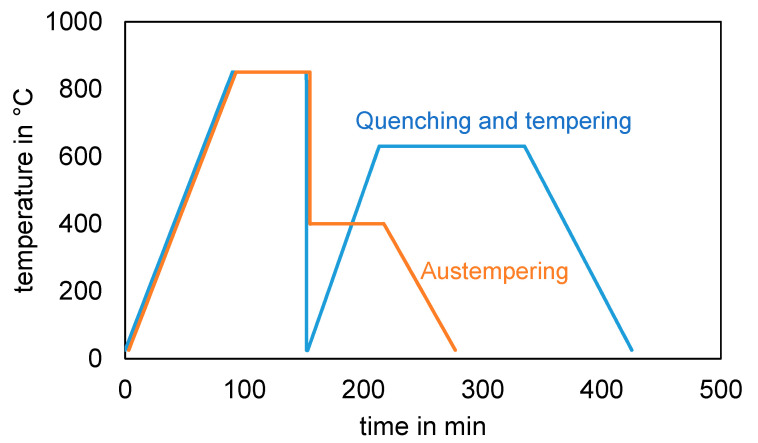
Comparison of the time-temperature curves of the preheating treatments quenching and tempering and austempering.

Figure 4 shows a comparison of the time-temperature curves for the two pre-heat treatments, quenching and tempering (QT) and austempering (OSA). The scematic diagram shows a clear time advantage for austempering because, on the one hand, the duration of the isothermal transformation is shorter than usual tempering times (2–3 h) and; on the other hand, quenching to room temperature and subsequent reheating is not necessary after austenitizing. The latter is therefore also associated with energy savings.

**Figure 5:**
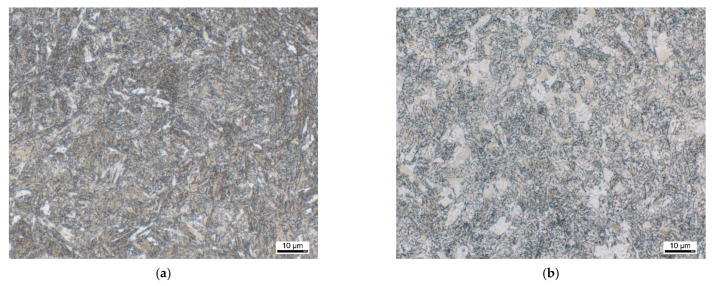

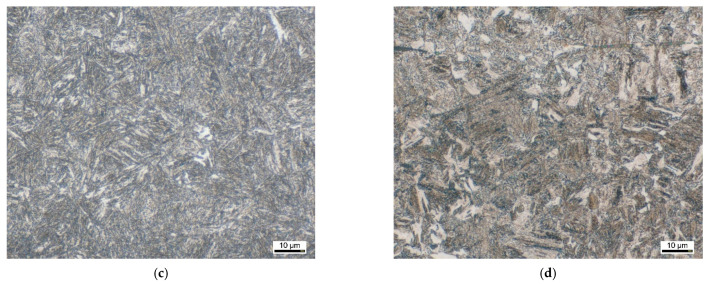
Light microscopic documentation of the microstructures after the pre-heat treatments quenching and tempering and austempering: (**a**) EN31CrMoV9, quenched and tempered; (**b**) EN31CrMoV9, austempered; (**c**) EN42CrMo4, quenched and tempered; (**d**) EN42CrMo4, austempered.

**Figure 6:**
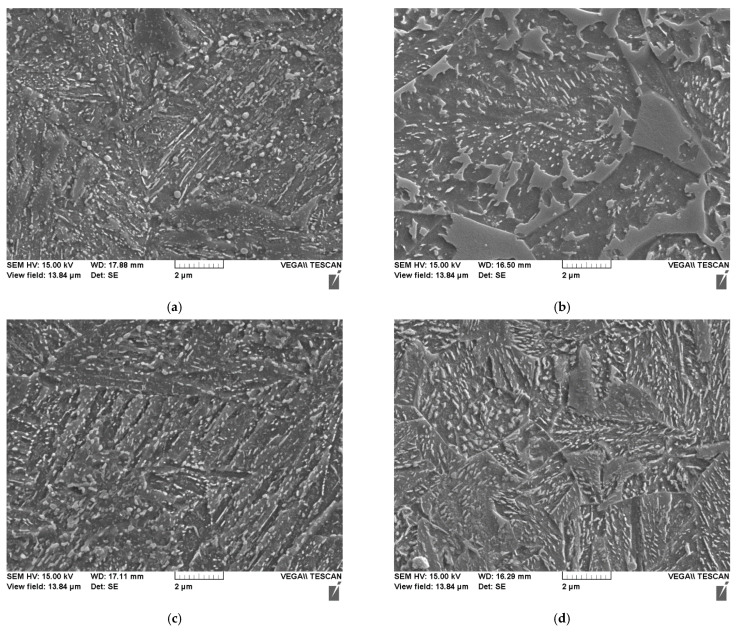
Scanning electron microscope documentation of the microstructures after the pre-heat treatments quenching and tempering and austempering: (**a**) EN31CrMoV9, quenched and tempered; (**b**) EN31CrMoV9, austempered; (**c**) EN42CrMo4, quenched and tempered; (**d**) EN42CrMo4, austempered.

The microstructures derived from pre-heat treatment after quenching and tempering and austempering of the investigation materials are shown with some light optical metallographical images in Figure 5 and Figure 6. After quenching and tempering, a fine, acicular quenched and tempered microstructure of tempered martensite is present. The lower bainite, formed during austempering, appears rather globular morphologically on a mesoscopic viewing plane and somewhat coarser concerning the carbide size and distribution than the tempered martensite. Because of the repeated temperature hysteresis with transformations into austenite and back a refinement of the microstructure was achieved. Both materials show a grain size of about 30 µm.

A specific feature visible in Figure 6b needs to be discussed further. The high resolution images taken by SEM exhibit some island like uprising structures showing nearly no substructure with carbides. These islands often are associated with martensite-austenite islands. In the case of the austempered sample, these islands are most likely a result of the positive segregations, which impact the transformation time and have caused an incomplete transformation of the EN31CrMoV9during soaking for 1 h in the austempering stage. Due to the lack of further tempering steps after cooling to room temperature, these islands have not been tempered after cooling and therefore do not show an etching relief. The very light substructure of the surface of these islands is indicating a martensite microstructure in non-tempered state. To avoid such remainders of martensite a two-step-austempering treatment or an elongated one-step-austempering treatment could be used.

**Figure 7:**
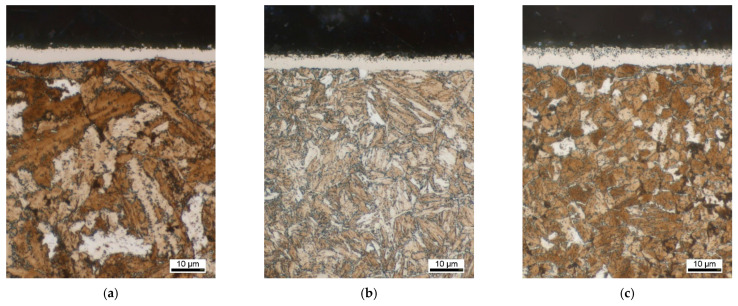

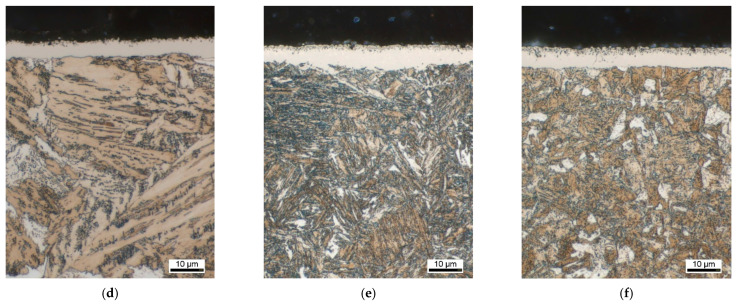
Light microscopic documentation of the compound layers after nitriding of the different pre-heat treatment conditions: (**a**) EN31CrMoV9 without pre-heat treatment; (**b**) EN31CrMoV9 quenched and tempered; (**c**) EN31CrMoV9 austempered; (**d**) EN42CrMo4 without pre-heat treatment; (**e**) EN42CrMo4 quenched and tempered; (**f**) EN42CrMo4 austempered.

Figure 7 shows the highly magnified metallographic images of the surface layers after nitriding of the differently pre-heat treated materials. It can be seen that the pre-heat treatment condition seemingly are not impacting the compound layer thickness. On closer inspection, however, it is noticeable that in both the austempered material EN31CrMoV9 and the austempered EN42CrMo4 the porous part of the compound layer is somewhat more pronounced than in the quenched and tempered condition.

Since first-order phase transformation does not occur during nitriding due to the treatment temperature being below the AC1 temperature, the microstructures of the diffusion layer look similar to the pre-heat treated states. As before nitriding, the microstructure in the diffusion zone is very coarse in the initial non-targeted pre-heat treated state. The two materials quenched and tempered prior to nitriding have a fine-needled microstructure; the grains in the two ausempered specimens also appear fine, but the expression of the morphology is more globular.

**Figure 8:**
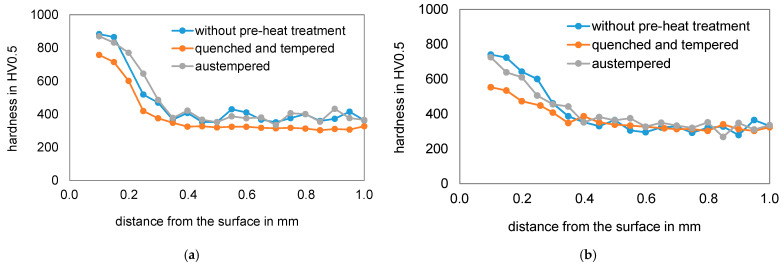
Hardness curves after nitriding of the different pre-heat treatment conditions: (**a**) EN31CrMoV9; (**b**) EN42CrMo4.

Figure 8 shows the hardness profiles measured on the metallographic cross section after nitriding of the two test materials with the three different pre-heat treatments. The bainitic initial microstructure seems to have a positive effect on the diffusion depth and hardness distribution. For both materials, a higher nitriding hardness depth is observed on the austempered specimens than on the quenched and tempered specimens. The maximum hardness is significantly higher on the specimens with the bainitic microstructure than of the quenched and tempered specimens. The specimens that were nitrided in the as-received condition each exhibit a hardness similar to that of the nitrided bainite. Towards the core, the difference in hardness between the initial microstructures becomes smaller after nitriding.

**Figure 9:**
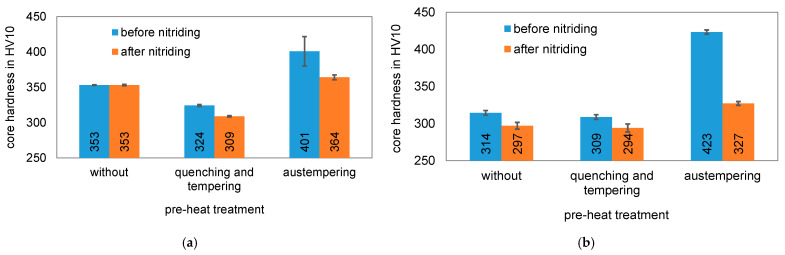
Comparison of core hardness before and after nitriding of the materials with different pre-heat treatment conditions: (**a**) EN31CrMoV9; (**b**) EN42CrMo4.

The core hardness of the differently pre-heat treated specimens before and after the nitriding process is compared in Figure 9. After austempering, the materials exhibit the highest core hardness. It is about 400 HV10 for the material EN31CrMoV9 and 420 HV10 for the material EN42CrMo4. The hardness after quenching and tempering is significantly lower, which can be attributed to the relatively high annealing temperature of 630 °C. By using a lower tempering temperature, a higher hardness can be set by quenching and tempering before nitriding. This could be the case with the as-delivered condition of EN31CrMoV9, as quenched and tempered steels are often already quenched and tempered in the steel manufacturing process and delivered with a certain strength. In the case of EN42CrMo4, a similar tempering temperature could have been used as for the quenched and tempered sample. Like the quenched and tempered conditions, the as-delivered conditions do not show any significant decrease in core hardness during nitriding. A significant decrease in core hardness is observed in the bainitized microstructure. Here, tempering takes place during nitriding, since the nitriding temperature of 520 °C is significantly higher than the austempering temperatures (400 °C for EN31CrMoV9 and 360 °C for EN42CrMo4). In the case of EN42CrMo4, where the difference between austempering and nitriding temperature is greater, the drop in core hardness is more pronounced than in the case of EN31CrMoV9.

## 4. Conclusions

Nitriding only achieves an increase in strength in the surface layer. In order to achieve a core strength that meets the requirements, it is necessary to subject the components to a preheating treatment before nitriding. However, a higher quench and temper strength also has a positive effect on the achievable strength in the nitrided surface layer. Since the mechanism of precipitation strengthening by the precipitated nitrides is effective in addition to the strength-increasing mechanisms due to the pre-heat treatment, higher strengths can be achieved in the nitrided layer if the material has a higher basic strength.

In practice, components to be nitrided are therefore frequently quenched and tempered. By increasing the austenitizing temperature for EN31CrMoV9, it was possible to achieve increased hardness due to finer precipitates being formed during tempering prior to nitriding compared to standard hardness parameters. The higher hardness was also retained after long-term tempering, so that a higher hardness can also be expected in the nitrided layer and core during subsequent nitriding.

Austempering also has potential as a pre-heat treatment for nitriding, since hardness values similar to those of tempered martensite can be achieved. Bainitic treatment instead of quenching and tempering promises time savings due to the shorter duration of the isothermal transformation compared to usual tempering durations of 2–3 h. Furthermore, austempering also means energy savings, because quenching to room temperature and subsequent reheating is not necessary after austenitizing as austempering is achieved by quenching to transformation temperature directly.

In the investigations presented above, the possibility of using a bainitic initial condition for nitriding of the two frequently used tempering and nitriding steels EN31CrMoV9 and EN42CrMo4 was investigated. The bainitic initial microstructure seems to have a positive effect on the diffusion depth, because a higher nitriding hardness depth is observed on the bainitized specimens than on the quenched and tempered specimens. In addition the maximum hardness at the beginning of the hardening curve (surface near) is significantly higher on the specimens with the bainitic microstructure than on the quenched and tempered specimens. The bainitic structures had also a higher core hardness than the quenched and tempered structures before nitriding. During subsequent nitriding, however, the bainite is tempered and loses some of its hardness. Whether and to what extent this results in dimensional and shape changes remains to be clarified.

## 5. Outlook

This study was only qualitative to show the potential of an optimized pre-heat treatment for nitriding. The investigation of the microstructure gave some clues that austempering could be a promising method for pre-heat treatment, but the technological properties of the nitrided bainite were not considered in this study. In the non-nitrided state, bainite generally exhibits higher ductility than martensite. During nitriding, ductility decreases due to precipitation of strength-enhancing nitrides, so the question remains open whether a bainitic pre-heat treatment structure has a positive effect on the ductility of the diffusion layer and performance of such treated parts. In this context, the microstructural changes, in particular the tempering of the bainite and the precipitation of the nitrides during the nitriding of the bainite, are to be further investigated and quantified by other techniques, i.e., physical methods.

One of the main applications of nitrided parts is the higher wear resistance. The study shows that the pre-heat treatment has an influence on the structure of the compound layer (especially the pores), which is the main reason for the high wear resistance of nitrided components. It is not known yet how austempering prior to nitriding influences the wear resistance of the nitrided layer.

Since the use of fluidized bed furnaces and salt baths to obtain bainite is not environmentally friendly, further studies should also concentrate on the possibilities to obtain bainite in alternative ways.

## Figures and Tables

**Table 1:** Overview of the heat treatment parameters.

Heat Treatment Stage	EN31CrMoV9	EN42CrMo4
Pre-heat Treatment Quenching and Tempering	870 °C 2 h/oil 60 °C 630 °C 2 h	850 °C 2 h/oil 60 °C 630 °C 2 h
	950 °C 2 h/oil 60 °C 630 °C 2 h	-
Pre-heat Treatment Austempering	850 °C 45 min/400 °C 1 h	850 °C 45 min/360 °C 1 h
Nitriding	520 °C 20 h K_N_ = 0.8	520 °C 20 h K_N_ = 0.8

**Table 2:** Heat treatment parameters for long-term tempering.

No.	1	2	3	4	5
Temperature in °C	520	530	550	570	590
Time in h	180	170	150	130	75

## Data Availability

The raw/processed data required to reproduce these findings cannot be shared at this time as the data also forms part of an ongoing study.
